# Supplementary material for: The Variations’ in Genes Encoding TIM-3 and Its Ligand, Galectin-9, Influence on ccRCC Risk and Prognosis
Source: Int J Mol Sci. 2023 Jan 20;24(3):2042. doi: 10.3390/ijms24032042 (PMC9917084; doi:10.3390/ijms24032042)
Supplement: Supplementary file 1 [file ijms-24-02042-s001.zip › Figure S1.pdf]

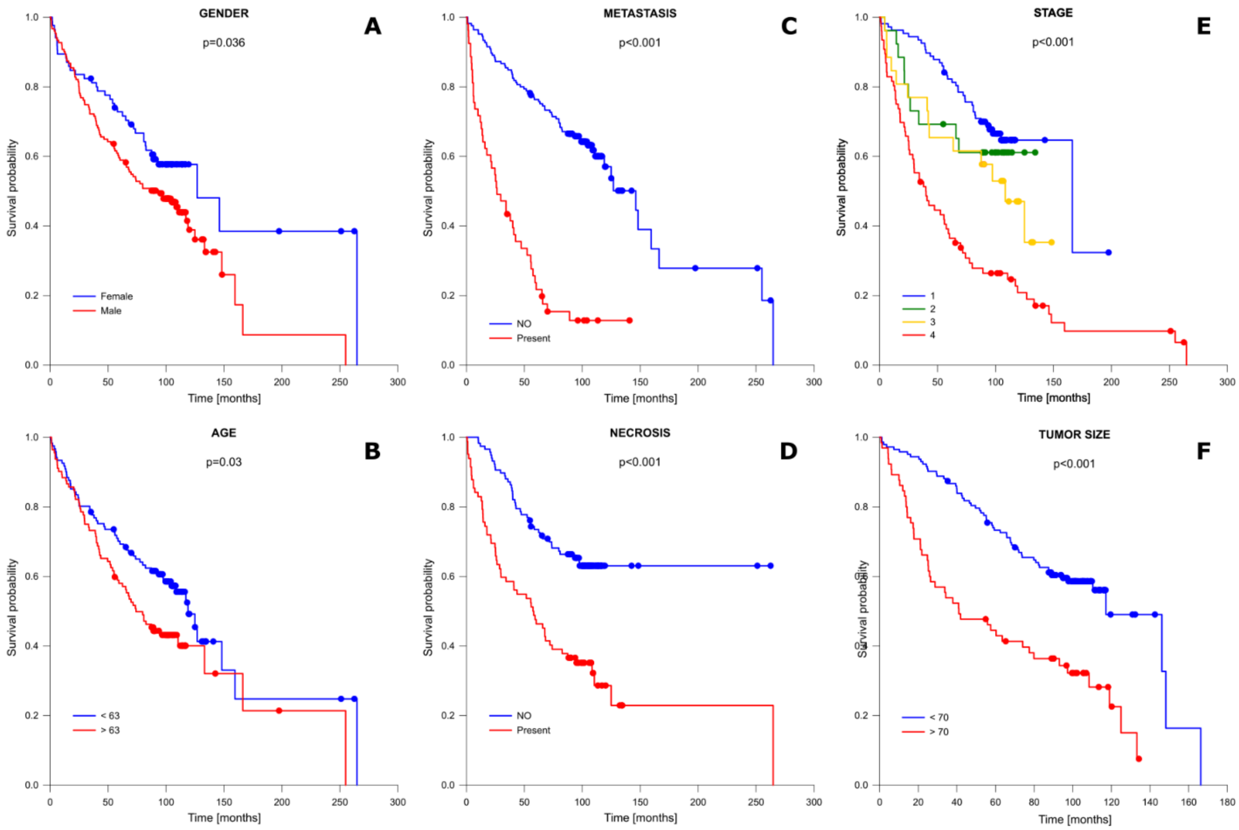

Figure S1 Probability of survival in relation to clinical features: **A**) gender; **B**) age (years); **C**) metastasis; **D**) necrosis; **E**) stage of disease; **F**) tumor size (in mm).
