# Supplementary material for: The Variations’ in Genes Encoding TIM-3 and Its Ligand, Galectin-9, Influence on ccRCC Risk and Prognosis
Source: Int J Mol Sci. 2023 Jan 20;24(3):2042. doi: 10.3390/ijms24032042 (PMC9917084; doi:10.3390/ijms24032042)
Supplement: Supplementary file 1 [file ijms-24-02042-s001.zip › Table S1.pdf]

Table S1 Genotype and allele distribution of *TIM-3* and *LGALS9* SNPs among male patients and male controls

			**Cases**		**Controls**					
SNP	Genotype	Allele	N	%	N	%	OR	95%CI		**p value**
**rs1036199**										
	AA		91	60.26	161	62.40	1			0.664
	AC		55	36.42	85	32.95	1.146	0.750	1.751	
	CC		5	3.31	12	4.65	0.777	0.276	2.187	
	AC+CC		60	39.74	97	37.60	1.095	0.726	1.651	0.668
	AA+AC		146	96.69	246	95.35	1.351	0.485	3.759	0.513
		A	237	78.48	407	78.88	1			
		C	65	21.52	109	21.12	1.026	0.727	1.450	0.893
**rs10057302**										
	CC		145	96.03	239	92.64	1			0.294
	AC		6	3.97	17	6.59	0.611	0.243	1.540	
	AA		0	0.00	2	0.78	0.329	0.016	6.906	
	AC+AA		6	3.97	19	7.36	0.549	0.221	1.365	0.168
	CC+AC		151	100.00	256	99.22	-	-	-	-
		C	296	98.01	495	95.93	1			
		A	6	1.99	21	4.07	0.505	0.208	1.229	0.108
**rs3751093**										
	GG		87	58.00	150	58.14	1			0.390
	AG		52	34.67	97	37.60	0.926	0.605	1.419	
	AA		11	7.33	11	4.26	1.720	0.729	4.060	
	AG+AA		63	42.00	108	41.86	1.007	0.670	1.512	0.978
	GG+AG		139	92.67	247	95.74	0.564	0.243	1.310	0.186
		G	226	75.33	397	76.94	1			
		A	74	24.67	119	23.06	1.094	0.785	1.525	0.603
**rs361497**										
	GG		81	54.00	139	54.51	1			0.753
	AG		58	38.67	102	40.00	0.977	0.641	1.489	
	AA		11	7.33	14	5.49	1.358	0.597	3.085	
	AG+AA		69	46.00	116	45.49	1.021	0.682	1.529	0.921
	GG+AG		139	92.67	241	94.51	0.728	0.327	1.623	0.457
		G	220	73.33	380	74.51	1			
		A	80	26.67	130	25.49	1.064	0.770	1.471	0.712
**rs4239242**										
	TT		61	40.40	115	44.57	1			0.682
	CT		69	45.70	112	43.41	1.160	0.755	1.784	
	CC		21	13.91	31	12.02	1.282	0.683	2.406	
	CT+CC		90	59.60	143	55.43	1.184	0.789	1.778	0.411
	TT+CT		130	86.09	227	87.98	0.840	0.466	1.515	0.580
		T	191	63.25	342	66.28	1			
		C	111	36.75	174	33.72	1.143	0.850	1.537	0.380
**rs4794976**										
	TT		74	49.01	126	48.84	1			0.319
	GT		61	40.40	115	44.57	0.904	0.593	1.378	
	GG		16	10.60	17	6.59	1.601	0.771	3.325	
	GT+GG		77	50.99	132	51.16	0.993	0.665	1.483	0.974
	TT+GT		135	89.40	241	93.41	0.595	0.294	1.204	0.152
		T	209	69.21	367	71.12	1			
		G	93	30.79	149	28.88	1.097	0.805	1.495	0.562
