# Supplementary material for: The Variations’ in Genes Encoding TIM-3 and Its Ligand, Galectin-9, Influence on ccRCC Risk and Prognosis
Source: Int J Mol Sci. 2023 Jan 20;24(3):2042. doi: 10.3390/ijms24032042 (PMC9917084; doi:10.3390/ijms24032042)
Supplement: Supplementary file 1 [file ijms-24-02042-s001.zip › Table S2.pdf]

Table S2 Genotype and allele distribution of *TIM-3* and *LGALS9* SNPs in patients above 63 years of age and controls

			Cases		Controls					
SNP	Genotype	Allele	N	%	N	%	OR	95%CI		**p value**
**rs1036199**										
	AA		69	57.02	256	62.44	1			0.290
	AC		49	40.50	137	33.41	1.329	0.874	2.021	
	CC		3	2.48	17	4.15	0.738	0.227	2.397	
	AC+CC		52	42.98	154	37.56	1.254	0.832	1.890	0.283
	AA+AC		118	97.52	393	95.85	1.506	0.469	4.832	0.398
		A	187	77.27	649	79.15	1			
		C	55	22.73	171	20.85	1.121	0.795	1.581	0.532
**rs10057302**										
	CC		114	94.21	376	91.71	1			-
	AC		7	5.79	32	7.80	0.759	0.334	1.725	
	AA		0	0.00	2	0.49	-	-	-	
	AC+AA		7	5.79	34	8.29	0.715	0.316	1.618	0.364
	CC+AC		121	100.00	408	99.51	-	-	-	-
		C	235	97.11	784	95.61	1			
		A	7	2.89	36	4.39	0.684	0.308	1.522	0.299
**rs3751093**										
	GG		68	56.67	242	59.02	1			**0.037**
	AG		38	31.67	147	35.85	0.924	0.592	1.442	
	AA		14	11.67	21	5.12	2.388	1.164	4.895	
	AG+AA		52	43.33	168	40.98	1.103	0.732	1.662	0.645
	GG+AG		106	88.33	389	94.88	0.405	0.201	0.816	**0.011**
		G	174	72.50	631	76.95	1			
		A	66	27.50	189	23.05	1.270	0.917	1.759	0.156
**rs361497**										
	GG		64	53.33	225	55.42	1			0.177
	AG		43	35.83	157	38.67	0.966	0.625	1.492	
	AA		13	10.83	24	5.91	1.926	0.938	3.957	
	AG+AA		56	46.67	181	44.58	1.088	0.724	1.635	0.687
	GG+AG		107	89.17	382	94.09	0.510	0.254	1.025	0.064
		G	171	71.25	607	74.75	1			
		A	69	28.75	205	25.25	1.198	0.870	1.651	0.277
**rs4239242**										
	TT		48	40.00	177	43.17	1			0.633
	CT		52	43.33	178	43.41	1.076	0.692	1.675	
	CC		20	16.67	55	13.41	1.352	0.743	2.458	
	CT+CC		72	60.00	233	56.83	1.136	0.752	1.717	0.537
	TT+CT		100	83.33	355	86.59	0.765	0.440	1.330	0.369
		T	148	61.67	532	64.88	1			
		C	92	38.33	288	35.12	1.150	0.855	1.547	0.362
**rs4794976**										
	TT		61	50.83	210	51.22	1			0.074
	GT		41	34.17	166	40.49	0.853	0.548	1.329	
	GG		18	15.00	34	8.29	1.835	0.975	3.454	
	GT+GG		59	49.17	200	48.78	1.016	0.677	1.524	0.941
	TT+GT		102	85.00	376	91.71	0.508	0.277	0.930	**0.030**
		T	163	67.92	586	71.46	1			
		G	77	32.08	234	28.54	1.186	0.870	1.616	0.289

Bolded values are significant.
