# Supplementary material for: The Variations’ in Genes Encoding TIM-3 and Its Ligand, Galectin-9, Influence on ccRCC Risk and Prognosis
Source: Int J Mol Sci. 2023 Jan 20;24(3):2042. doi: 10.3390/ijms24032042 (PMC9917084; doi:10.3390/ijms24032042)
Supplement: Supplementary file 1 [file ijms-24-02042-s001.zip › Table S4.pdf]

Table S4 Haplotype distribution of *TIM-3* SNPs between female patients and female controls

**Haplotype***	**ccRCC** (%)	**Control** (freq)	**Odds Ratio** [95%CI]	**p value**
A A	3.00 (1.7)	13.99 (4.7)	0.358 [0.101~1.264]	0.096
A C	141.00 (82.0)	220.01 (74.3)	1.571 [0.983~2.508]	0.058
C C	28.00 (16.3)	61.99 (20.9)	0.734 [0.449~1.201]	0.217
Global $\chi2$=4.72, df=2, p=0.095				

* rs1036199, rs10057302
