# Supplementary material for: The Variations’ in Genes Encoding TIM-3 and Its Ligand, Galectin-9, Influence on ccRCC Risk and Prognosis
Source: Int J Mol Sci. 2023 Jan 20;24(3):2042. doi: 10.3390/ijms24032042 (PMC9917084; doi:10.3390/ijms24032042)
Supplement: Supplementary file 1 [file ijms-24-02042-s001.zip › Table S5.pdf]

Table S5 Haplotype distribution of *LGALS9* SNPs between female patients and female controls

**Haplotype***	**ccRCC** (%)	**Control** (%)	**Odds Ratio** [95%CI]	**p value**
A A C G	49.91 (29.4)	58.03 (19.6)	1.678 [1.083~2.599]	**0.020**
A A C T	0.00 (0)	7.93 (2.7)	-	**0.030**
A G T T	2.05 (1.2)	1.01 (0.3)	3.498 [0.321~38.060]	0.273
G A C G	5.05 (3.0)	4.39 (1.5)	2.007 [0.551~7.316]	0.282
G G C G	4.58 (2.7)	9.92 (3.4)	0.788 [0.256~2.425]	0.677
G G C T	15.46 (9.1)	29.15 (9.8)	0.904 [0.473~1.727]	0.759
G G T G	7.46 (4.4)	9.66 (3.3)	1.344 [0.507~3.562]	0.551
G G T T	83.45 (49.1)	172.24 (58.2)	0.672 [0.459~0.983]	**0.040**
Global $\chi2$=17.08, df=7, p=**0.029**				

* rs3751093, rs361497, rs4239242, rs4794976. Bolded values are significant.
