# Supplementary material for: The Variations’ in Genes Encoding TIM-3 and Its Ligand, Galectin-9, Influence on ccRCC Risk and Prognosis
Source: Int J Mol Sci. 2023 Jan 20;24(3):2042. doi: 10.3390/ijms24032042 (PMC9917084; doi:10.3390/ijms24032042)
Supplement: Supplementary file 1 [file ijms-24-02042-s001.zip › Table S6.pdf]

Table S6 Probability of survival in relation to clinical features

Variable		Descriptive statistic				
		**Median**	**Mean**	**SD**	**Survived [n]**	**Death [n]**
**Gender**	Female	126.87	146.29	17.66	47	38
	Male	94.53	98.11	9.49	62	89
**Age**	< 63	119.03	127.99	13.95	63	58
	> 63	74.07	109.16	13.22	45	67
**Metastasis**	No	146.13	140.27	11.89	95	70
	Present	26.37	42.90	6.16	8	45
**Necrosis**	No	-	183.01	9.99	75	42
	Present	57.50	95.70	13.42	25	57
**Stage**	1	166.27	138.40	9.51	70	37
	2	-	93.72	10.86	16	10
	3	108.47	91.22	11.64	12	14
	4	39.87	70.57	9.42	11	65
**Tumor size**	< 70 mm	117.17	106.79	5.67	80	63
	> 70 mm	41.00	62.09	6.29	18	47
