# Supplementary material for: The Variations’ in Genes Encoding TIM-3 and Its Ligand, Galectin-9, Influence on ccRCC Risk and Prognosis
Source: Int J Mol Sci. 2023 Jan 20;24(3):2042. doi: 10.3390/ijms24032042 (PMC9917084; doi:10.3390/ijms24032042)
Supplement: Supplementary file 1 [file ijms-24-02042-s001.zip › Table S7.pdf]

Table S7 Probability of survival in relation to *TIM-3* and *LGALS9* genes polymorphisms

Variable		Descriptive statistic				
		**Median**	**Mean**	**SD**	**Survived [n]**	**Death [n]**
**rs1036199**	AA	124.97	122.74	11.80	76	72
	AC+CC	72.07	109.21	14.38	33	55
**rs10057302**	CC	108.47	117.01	9.44	105	122
	AC	79.83	76.55	12.53	5	4
**rs361497**	GG	117.17	108.41	12.65	58	62
	AA+AG	108.48	120.61	12.20	50	64
**rs4239242**	TT	97.63	10748.	14.95	42	49
	CC+CT	110.40	120.51	11.39	66	78
**rs479476**	TT	97.63	105.97	12.49	51	59
	GG+TG	110.40	122.50	12.17	57	68
**rs3751093**	GG	104.77	117.13	13.46	60	67
	AA+AG	108.47	113.54	12.91	47	59
