# Supplementary material for: The Variations’ in Genes Encoding TIM-3 and Its Ligand, Galectin-9, Influence on ccRCC Risk and Prognosis
Source: Int J Mol Sci. 2023 Jan 20;24(3):2042. doi: 10.3390/ijms24032042 (PMC9917084; doi:10.3390/ijms24032042)
Supplement: Supplementary file 1 [file ijms-24-02042-s001.zip › Table S8.pdf]

Table S8 Univariate and multivariate logistic regression analysis of risk factors' influence on ccRCC patients (versus control group)

		Univariate				Multivariate			
**Variables**		**OR**	**95 % CI**		**p-value**	**OR**	**95 % CI**		**p-value**
**rs1036199 (ref. AA)**	AC+CC	0.98	0.71	1.37	0.913	-	-	-	-
**rs10057302 (ref. CC)**	AC+AA	0.44	0.21	0.93	**0.031**	0.45	0.21	0.96	**0.039**
**rs3751093 (ref. GG)**	AG+AA	1.20	0.87	1.66	0.260	-	-	-	-
**rs361497 (ref. GG)**	AG+AA	1.19	0.86	1.64	0.287	-	-	-	-
**rs4239242 (ref. TT)**	CT+CC	1.21	0.87	1.68	0.252	-	-	-	-
**rs4794976 (ref. TT)**	GT+GG	1.20	0.87	1.66	0.259	-	-	-	-

Bolded values are significant.
