# Supplementary material for: The Variations’ in Genes Encoding TIM-3 and Its Ligand, Galectin-9, Influence on ccRCC Risk and Prognosis
Source: Int J Mol Sci. 2023 Jan 20;24(3):2042. doi: 10.3390/ijms24032042 (PMC9917084; doi:10.3390/ijms24032042)
Supplement: Supplementary file 1 [file ijms-24-02042-s001.zip › Table S9.pdf]

Table S9 Characteristics of *TIM-3* and *LGALS9* SNPs

**Position (GRCh38.p13)**	**Gene**	**SNP**	**Variation**	**Gene region**	**Genotyping method**
chr5:157104725	*TIM-3*	rs1036199	A>C,<br>R(Arg)>L(Leu)	Intron 3	TaqMan Assay<br>C___2082038_1_
chr5:157087601	*TIM-3*	rs10057302	C>A	Exon 6	TaqMan Assay<br>C__29607693_10
chr17:27631278	*LGALS9*	rs3751093	G>A,<br>G(Gly)>S(Ser)	Exon 1	ARMS PCR
chr17:27643607	*LGALS9*	rs361497	G>A,<br>G(Gly)>E(Glu)	Exon 5	RFLP PCR<br>BglI
chr17:27647232	*LGALS9*	rs4239242	T>C	Intron 8	RFLP PCR<br>EcoRI
chr17:27647519	*LGALS9*	rs4794976	T>G	Intron 9	TaqMan Assay<br>C__29024730_10
