# Supplementary material for: The Variations’ in Genes Encoding TIM-3 and Its Ligand, Galectin-9, Influence on ccRCC Risk and Prognosis
Source: Int J Mol Sci. 2023 Jan 20;24(3):2042. doi: 10.3390/ijms24032042 (PMC9917084; doi:10.3390/ijms24032042)
Supplement: Supplementary file 1 [file Table S3.pdf › Table S3.pdf]

**Table S3** Genotype and allele distribution of *TIM-3* and *LGALS9* SNPs depending on tumor size

| | | | **> 70 mm** | | **< 70 mm** | | | | | |
|---|---|---|---|---|---|---|---|---|---|---|
| SNP | Genotype | Allele | N | % | N | % | OR | 95% CI | | **p value** |
| **rs1036199** | | | | | | | | | | |
| | AA | | 38 | 58.46 | 90 | 62.94 | 1 | | | 0.768 |
| | AC | | 26 | 40.00 | 50 | 34.97 | 1.234 | 0.675 | 2.254 | |
| | CC | | 1 | 1.54 | 3 | 2.10 | 1.007 | 0.143 | 7.078 | |
| | AC+CC | | 27 | 41.54 | 53 | 37.06 | 1.208 | 0.667 | 2.190 | 0.540 |
| | AA+AC | | 64 | 98.46 | 140 | 97.90 | 1.071 | 0.155 | 7.419 | 0.786 |
| | | A | 102 | 78.46 | 230 | 80.42 | 1 | | | |
| | | C | 28 | 21.54 | 56 | 19.58 | 1.134 | 0.683 | 1.883 | 0.645 |
| **rs10057302** | | | | | | | | | | |
| | CC | | 59 | 90.77 | 140 | 97.90 | 1 | | | - |
| | AC | | 6 | 9.23 | 3 | 2.10 | 4.385 | 1.155 | 16.654 | |
| | AA | | 0 | 0.00 | 0 | 0.00 | - | - | - | |
| | AC+AA | | 6 | 9.23 | 3 | 2.10 | 4.385 | 1.155 | 16.654 | **0.019** |
| | CC+AC | | 65 | 100.00 | 143 | 100.00 | - | - | - | - |
| | | C | 124 | 95.38 | 283 | 98.95 | 1 | | | |
| | | A | 6 | 4.62 | 3 | 1.05 | 4.229 | 1.134 | 15.774 | **0.021** |
| **rs3751093** | | | | | | | | | | |
| | GG | | 32 | 49.23 | 79 | 56.03 | 1 | | | 0.580 |
| | AG | | 27 | 41.54 | 48 | 34.04 | 1.387 | 0.746 | 2.580 | |
| | AA | | 6 | 9.23 | 14 | 9.93 | 1.097 | 0.399 | 3.014 | |
| | AG+AA | | 33 | 50.77 | 62 | 43.97 | 1.311 | 0.730 | 2.354 | 0.364 |
| | GG+AG | | 59 | 90.77 | 127 | 90.07 | 1.041 | 0.393 | 2.760 | 0.875 |
| | | G | 91 | 70.00 | 206 | 73.05 | 1 | | | |
| | | A | 39 | 30.00 | 76 | 26.95 | 1.165 | 0.738 | 1.839 | 0.522 |
| **rs361497** | | | | | | | | | | |
| | GG | | 30 | 46.15 | 76 | 53.90 | 1 | | | 0.454 |
| | AG | | 29 | 44.62 | 50 | 35.46 | 1.465 | 0.789 | 2.719 | |
| | AA | | 6 | 9.23 | 15 | 10.64 | 1.052 | 0.384 | 2.881 | |
| | AG+AA | | 35 | 53.85 | 65 | 46.10 | 1.359 | 0.757 | 2.442 | 0.302 |
| | GG+AG | | 59 | 90.77 | 126 | 89.36 | 1.122 | 0.427 | 2.948 | 0.757 |
| | | G | 89 | 68.46 | 202 | 71.63 | 1 | | | |
| | | A | 41 | 31.54 | 80 | 28.37 | 1.166 | 0.744 | 1.829 | 0.512 |
| **rs4239242** | | | | | | | | | | |
| | TT | | 24 | 36.92 | 55 | 38.73 | 1 | | | 0.716 |
| | CT | | 31 | 47.69 | 60 | 42.25 | 1.179 | 0.621 | 2.240 | |
| | CC | | 10 | 15.38 | 27 | 19.01 | 0.865 | 0.368 | 2.034 | |
| | CT+CC | | 41 | 63.08 | 87 | 61.27 | 1.074 | 0.588 | 1.962 | 0.804 |
| | TT+CT | | 55 | 84.62 | 115 | 80.99 | 1.259 | 0.577 | 2.745 | 0.528 |
| | | T | 79 | 60.77 | 170 | 59.86 | 1 | | | |
| | | C | 51 | 39.23 | 114 | 40.14 | 0.965 | 0.632 | 1.473 | 0.861 |
| **rs4794976** | | | | | | | | | | |
| | TT | | 29 | 44.62 | 67 | 47.18 | 1 | | | 0.428 |
| | GT | | 29 | 44.62 | 52 | 36.62 | 1.286 | 0.688 | 2.402 | |
| | GG | | 7 | 10.77 | 23 | 16.20 | 0.730 | 0.288 | 1.849 | |
| | GT+GG | | 36 | 55.38 | 75 | 52.82 | 1.106 | 0.616 | 1.987 | 0.732 |
| | TT+GT | | 58 | 89.23 | 119 | 83.80 | 1.534 | 0.637 | 3.696 | 0.304 |
| | | T | 87 | 66.92 | 186 | 65.49 | 1 | | | |
| | | G | 43 | 33.08 | 98 | 34.51 | 0.941 | 0.607 | 1.459 | 0.776 |

Bolded values are significant.
